# Kidney Involvement in SARS-CoV-2 Infection: Peritoneal Dialysis as the Preferred Modality

**DOI:** 10.3390/vaccines13070723

**Published:** 2025-07-02

**Authors:** Marko Baralić, Nikola Stojanović, Selena Gajić, Aleksandar Sič, Aarish Manzar, Ana Bontić, Jelena Pavlović, Mateja N. Bojić, Aleksandra Kezić

**Affiliations:** 1 Faculty of Medicine, University of Belgrade, Dr. Subotića Starijeg Str. 8, 11000 Belgrade, Serbiaaca.smed01@gmail.com (A.S.); mateja.bojic2600@gmail.com (M.N.B.); aleksandra.kezic@med.bg.ac.rs (A.K.); 2 Department of Nephrology, University Clinical Centre of Serbia, Pasterova Str. 2, 11000 Belgrade, Serbia; 3 Department of Physiology, Faculty of Medicine, University of Niš, Bulevar Dr. Zorana Đinđića Str. 81, 18000 Niš, Serbia; 4 Department of Internal Medicine, Ziauddin Medical College, Karachi 75500, Pakistan

**Keywords:** SARS-CoV-2, peritoneal dialysis, immune response, vaccines, chronic kidney disease, acute kidney injury, inflammation markers, cytokine response

## Abstract

Patients undergoing peritoneal dialysis (PD) represent a uniquely vulnerable population due to intrinsic immunological dysfunction and a high prevalence of comorbid conditions. This review examines the complex interplay between natural and vaccine-induced immune responses to SARS-CoV-2 in this group, focusing on viral entry, immune activation, and immune evasion mechanisms. Particular attention is given to the impaired cellular and humoral responses seen in PD patients, including reduced T-cell function, diminished antibody production, and abnormal cytokine signaling, all of which contribute to an elevated risk of severe COVID-19 outcomes. The immunogenicity and clinical efficacy of various vaccine platforms, including inactivated, vector-based, and mRNA formulations, are critically assessed, with an emphasis on the role of booster doses in enhancing protection amid waning immunity and evolving viral variants. Furthermore, the review highlights the advantages of PD as a home-based modality that is compatible with telemedicine and may reduce the risk of viral exposure. These insights underscore the importance of developing individualized vaccination strategies, maintaining close immunological surveillance, and implementing innovative dialysis care approaches to improve clinical outcomes during the ongoing pandemic and future public health crises. Tailored booster strategies and telemedicine-integrated care models are essential for improving outcomes in this high-risk population.

## 1. Introduction

SARS-CoV-2 is an enveloped, single-stranded RNA virus from the Coronaviridae family. It encodes four major structural proteins—Spike (S), Envelope (E), Membrane (M), and Nucleocapsid (N)—as well as several non-structural proteins involved in immune evasion [1,2,3]. Viral entry is mediated by the angiotensin-converting enzyme 2 (ACE2) receptor, which is highly expressed in type II pneumocytes, myocardial tissue, intestinal enterocytes, and renal proximal tubules [4,5]. This receptor distribution underlies the virus’s capacity to cause both acute kidney injury and exacerbate chronic kidney disease (CKD) [6,7]. Entry is further facilitated by the host serine protease TMPRSS2, which is co-expressed with ACE2 on epithelial cells in the respiratory tract, kidneys, and gastrointestinal system. TMPRSS2 cleaves the S protein at the S1/S2 junction, enabling conformational changes that allow membrane fusion and viral internalization [7,8].

In immunocompetent individuals, the immune response to SARS-CoV-2 involves both innate and adaptive arms. Neutralizing antibodies targeting the S protein appear early; however, data on other immunoglobulin classes and long-term cellular responses remain limited [9,10]. Viral entry activates innate immunity, including interferon (IFN) production, which contributes to systemic symptoms such as fever and myalgia [11,12,13]. SARS-CoV-2 proteins stimulate monocytes and macrophages, eliciting robust inflammatory responses [14,15]. The E protein activates Toll-like receptors (TLRs), enhancing cytokine release via neutrophil proteins like S100A8/A9 [16]. The S protein also interacts with C-type lectin receptors and mannose-binding lectin, leading to an increased production of proinflammatory cytokines such as IL-6, IL-8, and TNF-α [17,18,19]. Immune complexes involving the N protein and IgG can activate the NLRP3 inflammasome, releasing IL-1β and IL-18 and triggering pyroptosis [20]. Moreover, TNF-α and IFN-γ contribute to apoptotic death via FADD and caspase-8, culminating in PANoptosis—a highly inflammatory form of cell death [21]. A cytokine storm marked by elevated IL-6, IL-10, and TNF-α is associated with ARDS and multi-organ failure [22]. Viral non-structural proteins also interfere with IFN signaling, dampening antiviral responses and exacerbating disease severity [23]. These effects are likely amplified in immunocompromised populations, such as patients undergoing peritoneal dialysis [23].

Individuals on peritoneal dialysis (PD) exhibit altered immune responses, including impaired T cell activation and diminished antibody production, potentially undermining vaccine effectiveness [10,11,22]. These immunological impairments, combined with comorbidities like diabetes and cardiovascular disease, contribute to worse clinical outcomes following SARS-CoV-2 infection. Evidence-based strategies shown to improve prognosis in this group include timely booster vaccinations, the prophylactic use of monoclonal antibodies in high-risk individuals, early antiviral therapy and stringent infection control practices adapted to dialysis settings [9,11].

This review explores the relationship between natural and vaccine-induced immunity against SARS-CoV-2, focusing on patients undergoing peritoneal dialysis. This group shows unique immunological vulnerabilities due to impaired cellular and humoral responses, altered cytokine profiles, and common comorbidities. It integrates mechanisms of innate and adaptive immunity, including T and B lymphocytes, memory formation, antibody production and viral immune evasion. It also examines how vaccination influences immune protection and response durability in dialysis patients, and the impact on clinical outcomes such as infection severity and mortality. By combining insights from immunology, nephrology and virology, this review supports targeted immunization protocols, clinical monitoring, and infection prevention for this high-risk population.

## 2. Immunity

### 2.1. Cellular Immunity

SARS-CoV-2 exits host cells via the lytic pathway, triggering cellular immune responses primarily through CD4^+^ T-helper cells and natural killer (NK) cells, which in turn activate CD8^+^ cytotoxic T lymphocytes and promote B-cell-mediated antibody production [24]. The majority of infected individuals (70–100%) develop detectable T-cell responses within 1–2 weeks, even in the absence of a strong humoral response. However, SARS-CoV-2 infection often results in T-cell depletion and functional exhaustion, leading to lymphopenia—a consistent laboratory marker associated with severe disease, an elevated viral load, and increased mortality risk [25,26].

The upregulation of exhaustion markers such as PD-1 and Tim-3 on T cells reflects impaired antiviral immunity and correlates with poor clinical outcomes, particularly in critically ill patients [22]. Despite this, memory T-cell responses are often durable, and cross-reactive T cells from previous coronavirus infections may provide partial immunity in some individuals [27]. The viral invasion of alveolar type II pneumocytes triggers damage-associated molecular pattern (DAMP) signaling, activating macrophages and recruiting additional immune cells, ultimately resulting in vascular damage, pulmonary edema and acute respiratory distress syndrome (ARDS). Histopathological findings include hyaline membrane formation, epithelial cell debris, and monocyte/macrophage infiltration [28,29].

The broad expression of ACE2 receptors in epithelial tissues, including the lungs, kidneys, gastrointestinal tract, myocardium and vasculature, accounts for the frequent multi-organ involvement observed in COVID-19 [5,30]. This receptor distribution permits direct viral invasion and damage across multiple organ systems, contributing to systemic inflammation, endothelial dysfunction, and complications such as acute kidney injury, myocarditis, and gastrointestinal symptoms [5,30].

### 2.2. Humoral Immunity

B-cell-mediated antibody production is a central component of the host’s defense against SARS-CoV-2 infection [31,32]. The humoral immune response proceeds sequentially with the production of immunoglobulin M (IgM), immunoglobulin A (IgA), and immunoglobulin G (IgG). IgM appears first and offers immediate but transient protection, serving as an early marker of acute infection. IgA follows soon after and is particularly critical for mucosal immunity in the respiratory and gastrointestinal tracts, where it neutralizes the virus at the portals of entry. IgG appears later and provides longer-term systemic protection through viral neutralization and antibody-dependent cellular cytotoxicity [33]. This immunoglobulin cascade reflects a well-orchestrated transition from early containment to long-term immune memory.

Seroconversion typically occurs between 6 and 10 days following symptom onset. Although circulating antibody titers may wane within 6 to 13 months, memory B cells persist for over a year, enabling rapid secondary immune responses upon re-exposure [30,34,35]. However, newly emerging viral variants, particularly the Omicron lineage, demonstrate greater immune escape potential, with reinfection rates increasing from approximately 2.7% during the Delta wave to nearly 28.8% during the Omicron wave [36].

**Figure 1:**
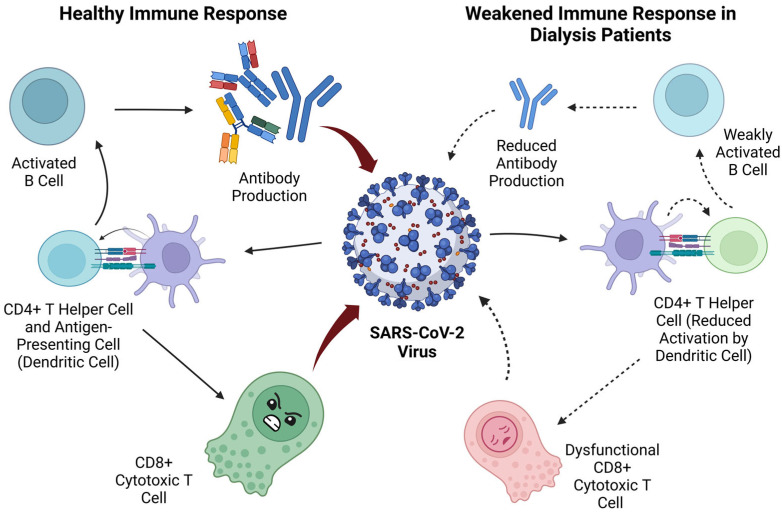
The differences in immune response between healthy individuals and dialysis patients infected with SARS-CoV-2.

These immunological dynamics are especially relevant in patients undergoing dialysis, who often exhibit immune exhaustion, reduced T-cell activation, and impaired antibody production. This immunodeficiency is driven by chronic inflammation, the accumulation of uremic toxins and repeated antigenic stimulation, collectively limiting both the magnitude and quality of humoral responses following infection or vaccination [11,22]. Consequently, vaccine effectiveness is diminished and susceptibility to reinfection is increased in this population. Figure 1 illustrates these differences, comparing the immune responses of healthy individuals and dialysis patients, and highlighting the reduced cellular and humoral immunity observed in the latter.

## 3. SARS-CoV-2 Vaccination

### 3.1. SARS-CoV-2 Vaccine Platforms and Effectiveness

Vaccination against SARS-CoV-2 elicits a robust and durable immune response, primarily by stimulating the production of neutralizing antibodies targeting the viral Spike (S) protein [37]. Various vaccine platforms have been developed, differing in antigen composition and delivery mechanisms [38].

Inactivated vaccines, such as Sinopharm, contain whole viral particles rendered non-infectious, allowing antigen-presenting cells (APCs) to process multiple viral components, including the S protein. Although considered safe, their immunogenicity may be suboptimal, and the optimal dosing regimen remains uncertain [39,40].

Vector-based vaccines, including Sputnik V and AstraZeneca, utilize recombinant adenoviral vectors encoding the S protein gene. While effective, these vaccines have been associated with rare hypersensitivity reactions, possibly due to excipients such as polysorbate 80 or EDTA, which can activate complement or mast cells in predisposed individuals, leading to non-IgE-mediated anaphylactoid reactions [41,42].

mRNA vaccines (Pfizer-BioNTech and Moderna) employ lipid nanoparticles to deliver synthetic messenger RNA encoding the S protein. Despite being mainly composed of naturally occurring substances, they may provoke rare allergic reactions, particularly to polyethylene glycol (PEG), a polymer used to stabilize the lipid nanoparticle shell, which may act as a hidden allergen in sensitized individuals [43]. Large-scale clinical trials have shown that the efficacy of mRNA vaccines in preventing symptomatic infection is approximately 95% [44], while real-world data confirm substantial reductions in severe disease and mortality among vaccinated individuals. However, vaccine-induced immunity wanes over time, particularly in vulnerable populations [45].

The observed decline in antibody titers and increased rates of breakthrough infections underscore the need for booster vaccinations. Booster doses restore waning immunity, especially in high-risk individuals, and are vital for maintaining adequate protection against emerging variants. Protection against severe illness generally persists despite declining neutralizing antibody levels. Some studies report breakthrough infections in approximately 2% of vaccinated individuals, emphasizing that although infrequent, such cases may still occur [46].

Comparative data from Bahrain, where four vaccines were deployed (Sinopharm, Sputnik V, Pfizer-BioNTech, and AstraZeneca), revealed considerable variability in efficacy. Sinopharm exhibited the lowest performance, while Pfizer-BioNTech provided the highest protection, with a 2- to 25-fold lower risk of infection compared to Sinopharm [47].

While most vaccines maintained strong efficacy against the original SARS-CoV-2 strain (Alpha variant), effectiveness against Beta, Gamma, and Delta variants has varied. Protection against the Beta variant was notably reduced, although supporting data remain limited [48]. A U.S. veteran study reported that vaccine effectiveness against the Delta variant one month post-vaccination declined to 58% for Moderna, 43% for Pfizer-BioNTech, and 13% for Johnson & Johnson. Protection against mortality also varied by age: among individuals under 65 years, vaccine-associated mortality protection was 82% for Moderna, 84% for Pfizer-BioNTech, and 73% for J&J, while in those over 65 years, it declined to 76%, 70%, and 52%, respectively [49,50].

Since the emergence of Omicron, vaccine effectiveness against mild or asymptomatic infection has declined further due to ongoing viral evolution [36]. Nonetheless, vaccines remain highly effective in preventing severe outcomes such as hospitalization and death. To address immune escape by emerging subvariants like XBB.1.5, variant-adapted booster formulations are being developed and recommended. These updated vaccines target conserved regions of the virus or incorporate antigens from circulating variants to sustain population-level immunity [45].

### 3.2. Booster Doses

Despite high vaccine coverage, many countries have experienced renewed SARS-CoV-2 outbreaks and introduced booster recommendations, initially targeting high-risk populations and later expanding to the general public in some regions (Serbia, Bahrain, and Hungary) [51,52]. Heterologous (mix and match) booster regimens have demonstrated both safety and efficacy, with mRNA vaccines providing the highest immunogenicity following inactivated or vector-based primary schedules [53].

The enhanced antibody response observed with heterologous regimens is thought to result from the broader stimulation of the immune system through multiple antigen delivery pathways. Inactivated vaccines may prime the immune system by exposing it to the whole virus, while mRNA boosters strongly amplify Spike-specific responses by directly enhancing antigen expression in host cells. This complementary stimulation leads to more diverse B and T cell activation and higher antibody titers [54].

In Israel, administering a third dose was associated with a 93 percent reduction in COVID-19-related hospitalizations [55]. During the Omicron wave, studies demonstrated that vaccine effectiveness against infection increased from approximately 21 percent after two doses to around 62 percent following a third dose [56].

The three-dose strategy was first explored during Ebola vaccine trials to extend the duration of protection in high-risk regions and optimize immune memory. The rationale was based on priming the immune system with an initial series, followed by a delayed booster to elicit durable long-lived B cell and T cell memory. This model translated to COVID-19 vaccine efforts, particularly in the context of emerging variants and waning immunity, offering a template for enhancing population-level immunity during outbreaks [57,58].

The type of initial vaccine platform plays a critical role in shaping booster effectiveness. Individuals primed with inactivated vaccines (e.g., SinoVac) often exhibit weaker neutralizing responses compared to those receiving mRNA vaccines. When an mRNA booster is administered following inactivated vaccine priming, it induces significantly stronger neutralizing antibody levels and cellular immunity compared to homologous boosting [54]. For instance, a study in Turkey reported a 46.6-fold increase in anti-Spike antibody levels after administering a Pfizer-BioNTech booster to individuals previously immunized with SinoVac [59].

Immune responses may also differ by dialysis modality. Compared to the general population, HD patients often show lower or delayed antibody responses, while PD patients may demonstrate more preserved T-cell responses due to reduced systemic inflammation. Nonetheless, comparative data are limited and individualized vaccination strategies remain essential [60].

### 3.3. Vaccination Recommendations for High-Risk Comorbidities

Vaccination is strongly recommended for individuals with chronic comorbidities, including patients with malignancies, cerebrovascular disease (stroke), CKD (especially those on dialysis), chronic pulmonary and liver disease, cardiovascular disease (CVD), hypertension (HTN), diabetes mellitus (DM) type I and II, psychiatric disorders, obesity, pregnancy, a history of smoking, tuberculosis, and immunodeficiency states [61]. These high-risk groups are more susceptible to severe COVID-19 outcomes and benefit significantly from immunization. For example, among dialysis patients, mortality decreased from approximately 29.5% during the pre-vaccination era to 6.7% during a post-vaccination wave [62].

However, immunization among dialysis patients poses specific challenges. Chronic systemic inflammation, persistent uremia and impaired antigen presentation contribute to suboptimal T- and B-cell responses, reducing vaccine-induced immunity. Additionally, logistic barriers such as timing doses around dialysis sessions and increased frailty, may further complicate vaccine delivery and monitoring. Therefore, dialysis patients often require booster regimens, individualized vaccination schedules and post-vaccination serological monitoring to ensure sufficient protection [60].

Vaccination during pregnancy is also crucial, as SARS-CoV-2 infection increases the risk of preterm birth, preeclampsia, and maternal complications. mRNA vaccines have demonstrated safety in pregnancy and are associated with the transfer of IgG antibodies across the placenta, conferring passive immunity to the newborn. Guidelines now recommend immunization at any stage of pregnancy, with no increased risk of adverse fetal outcomes [63].

Patients with psychiatric disorders face unique barriers to immunization, including vaccine hesitancy, reduced access to healthcare, and cognitive impairment affecting consent. Studies have shown that vaccine coverage is significantly lower in individuals with severe mental illness. Strategies to improve uptake include targeted education, mobile vaccination clinics and integrating vaccine administration into psychiatric care settings [64].

Overall, vaccination markedly improves clinical outcomes in individuals with chronic conditions [65].

### 3.4. Complications of SARS-CoV-2 Vaccines

The most common side effects of COVID-19 vaccines include localized pain at the injection site (79–86%), fatigue (60–66%), and headache (55–65%) [66]. Rare but clinically significant adverse events include myocarditis and pericarditis, particularly in males under 30 years of age, with incidence rates of up to 40 per million after the second dose of an mRNA vaccine. These cases are generally mild and rarely require intensive care [67,68].

Thrombosis with thrombocytopenia syndrome (TTS) has been linked to adenoviral vector vaccines, most notably ChAdOx1 nCoV-19. Epidemiological data suggest a higher risk in women under 50 years of age, potentially due to immunologic and hormonal factors that remain incompletely understood. In this demographic, TTS incidence has ranged from 2.3 to 8.1 per million in Europe and approximately 3 per million in the United States, with case fatality rates declining from an initial 50% to 15–20% due to improved recognition and early intervention [69,70].

Active pharmacovigilance systems VAERS (United States) and EudraVigilance (European Union) continue to provide real-time safety monitoring and ensure transparency. According to the European Medicines Agency, the overall safety profile of approved vaccines remains favorable, with serious adverse events occurring only in extremely rare cases [71,72].

Risk mitigation strategies include the preferential use of mRNA vaccines in high-risk groups and avoiding adenoviral vector vaccines unless clinically indicated. Screening for thrombotic risk factors and educating patients on early warning signs (headache, visual disturbances, dyspnea) are also recommended [73].

Anaphylaxis is another rare complication, occurring in 0.5–1.5 cases per million doses, often attributed to excipients such as polyethylene glycol (in mRNA vaccines), polysorbate 80 (in vector vaccines), or aluminum (in inactivated vaccines) [74,75]. Proposed mechanisms include IgE-mediated hypersensitivity and complement activation [75,76,77].

In confirmed cases of excipient allergy, referral to an allergist for skin testing and risk stratification is recommended. If allergy is confirmed, re-vaccination with the same formulation is contraindicated and alternative vaccines that lack the offending component should be considered. In patients with unrelated or mild allergic histories, a 30 min observation period post-vaccination is generally considered sufficient [78].

## 4. Chronic Kidney Disease in SARS-CoV-2 Infection

**Figure 2:**
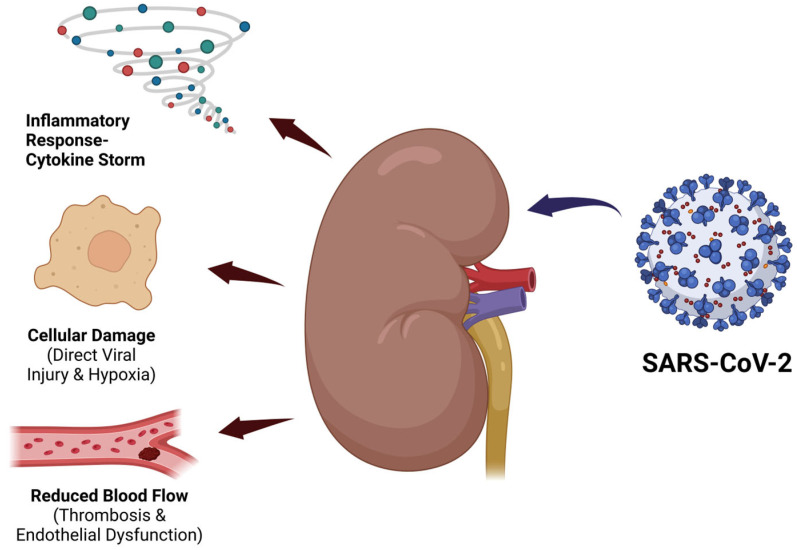
Pathways of SARS-CoV-2-induced renal injury.

Although SARS-CoV-2 was initially identified as a respiratory pathogen, clinical evidence has revealed its strong tropism for the kidneys, particularly in CKD patients. CKD has emerged as an important comorbidity influencing the clinical course of SARS-CoV-2 infection. Patients with pre-existing CKD are predisposed to worsened renal function and adverse outcomes due to a combination of direct viral effects and systemic inflammatory responses [79]. SARS-CoV-2 targets renal cells by binding to the ACE2 receptor, highly expressed in proximal tubular epithelial cells and podocytes, facilitating viral entry and potential cytotoxicity [7]. In addition to this direct invasion, the chronic inflammatory state characteristic of CKD amplifies cytokine-mediated tissue injury during COVID-19, contributing to progressive renal impairment [80]. Proinflammatory cytokines IL-6 and TNF-α are major mediators of this process. Elevated IL-6 levels correlate with disease severity and promote endothelial activation, increased vascular permeability and immune cell infiltration in renal tissue. TNF-α contributes to renal apoptosis and fibrosis, further exacerbating kidney damage [17,18,22]. The multifactorial pathways of SARS-CoV-2-induced renal injury, including direct viral cytotoxicity, cytokine storm, endothelial dysfunction and microvascular thrombosis, are illustrated in Figure 2.

Recent clinical studies have shown that individuals with CKD who recover from COVID-19 experience accelerated declines in kidney function compared to those with other respiratory infections, indicating a SARS-CoV-2-specific impact on renal health [81]. This accelerated decline is likely multifactorial, involving immune dysregulation, endothelial dysfunction and prothrombotic states that exacerbate microvascular injury in renal tissues [82]. Moreover, altered immune responses in CKD patients, including impaired viral clearance and persistent inflammation, may contribute to prolonged disease and renal damage [83]. Specific biomarkers and histopathological features have been identified in COVID-19-associated kidney ischemia, including acute tubular injury with endothelial swelling, microvascular thrombosis, and peritubular capillary rarefaction. Soluble thrombomodulin and von Willebrand factor indicate endothelial damage, while urinary neutrophil gelatinase-associated lipocalin (NGAL) reflects tubular injury, underscoring the unique thromboinflammatory mechanisms in SARS-CoV-2 renal pathology [84].

Although variability exists in individual outcomes, the evidence underscores the need for comprehensive post-infection monitoring of renal function in CKD patients affected by COVID-19 [85]. Furthermore, specific genetic factors, such as high-risk APOL1 variants, have been implicated in COVID-19-associated collapsing glomerulopathy, a severe form of kidney injury predominantly affecting individuals of African descent. This highlights the role of host genetics in modulating renal susceptibility and disease severity in SARS-CoV-2 infection [86]. Enhanced surveillance and individualized therapeutic strategies aimed at mitigating inflammation and protecting residual kidney function are critical to prevent progression toward end-stage kidney disease [80].

## 5. Peritoneal Dialysis

Peritoneal dialysis (PD) is a widely used method of renal replacement therapy. It currently treats approximately 300,000 patients with end-stage kidney disease (ESKD) worldwide, which accounts for about 11% of the total dialysis population [87]. The technique relies on the principles of diffusion and osmosis, using the peritoneal membrane (PM) as a semi-permeable barrier for the exchange of solutes and water [87].

The PM lines the abdominal cavity and internal organs and consists of a layer of mesothelial cells (simple squamous epithelium), a submesothelial interstitium with connective tissue, glycosaminoglycan-rich matrix, and an underlying capillary network [88]. The capillary endothelium is the primary exchange site, with its structure and pore system determining transport efficiency [89]. According to the Three-Pore Model (Troelstra’s theory), transport occurs through (1) small pores (40–50 Å), which account for 99.5% of the pore surface and allow the diffusion of small solutes and water; (2) large pores (approximately 250 Å), responsible for the transcapillary movement of macromolecules like proteins and immunoglobulins; and (3) aquaporins (AQP-1), ultrasmall water channels (<3 Å) that permit pure water transport through osmosis [90].

Diffusion in PD enables the movement of small solutes such as urea and creatinine from blood to dialysate down their concentration gradients, while osmosis facilitates ultrafiltration (UF) using a hyperosmolar glucose-based dialysis solution [91,92]. A phenomenon known as “sodium sieving” reflects the initial rapid drop in the dialysate sodium concentration due to free water movement through aquaporins, followed by slower solute transport [93].

The most common complications of long-term PD treatment are potential exit site infections and peritonitis. After peritonitis, changes occur in the peritoneal membrane (PM), which can also be caused by the dialysis solution itself. This leads to an impaired ultrafiltration capacity and the accelerated transport of small solutes [94]. Unlike hemodialysis, which induces systemic immune activation through blood–membrane interactions, peritoneal dialysis elicits a localized immune response due to the continuous exposure of the peritoneal membrane to dialysis solutions. This results in chronic peritoneal inflammation involving the activation of resident immune cells, contributing to membrane fibrosis and altered immune signaling unique to PD patients [95].

The early detection of PM dysfunction can be facilitated by biomarkers such as matrix metalloproteinase-2 (MMP-2) and tissue inhibitor of metalloproteinase-1 (TIMP-1), which are linked to peritoneal fibrosis. Elevated soluble CD59 levels and low circulating α-Klotho are also associated with membrane vulnerability. Metabolites like lactate and succinate may indicate peritoneal health, especially in pediatric patients. Increased peritoneal protein leakage also predicts a higher peritonitis risk [96]. NGAL in dialysate has shown promise as a reliable marker for early peritonitis detection. Other immune markers such as tumor necrosis factor-like weak inducer of apoptosis (TWEAK) and prothrombin fragment 1 + 2 (F1 + 2) in effluent reflect local inflammation and peritoneal permeability alterations, respectively [97].

The procedure involves instilling warmed dialysis fluid into the peritoneal cavity, where it remains for a prescribed dwell time. During this period, metabolic waste and excess fluid are removed through the capillary–peritoneal interface via diffusion and osmosis [98]. In addition to local effects on the peritoneum, systemic inflammation and comorbidities such as diabetes mellitus significantly influence peritoneal solute transport rates by increasing membrane permeability and causing microvascular changes. Diabetic patients typically exhibit higher solute transport rates, which can impair dialysis adequacy and require personalized treatment adjustments [99].

### 5.1. PD Treatment Modalities

Peritoneal dialysis (PD) can be delivered either as continuous ambulatory peritoneal dialysis (CAPD), performed manually through four daily exchanges, or as automated peritoneal dialysis (APD), conducted overnight using a cycler [100]. Individual peritoneal membrane transport rates guide the choice of modality, assessed via the Peritoneal Equilibration Test (PET), which classifies patients into fast, fast-average, slow-average, or slow transporters based on solute kinetics [101].

APD has gained popularity in high-income countries due to technological advances and the implementation of remote monitoring systems. It is at least as effective as CAPD in achieving dialysis targets, with advantages including lower peritonitis rates, an improved quality of life, and a 13% reduction in mortality, as demonstrated in a 2023 meta-analysis of over 230,000 patients. Remote telemetry further enhances APD management by enabling real-time monitoring and reducing the need for frequent in-person evaluations [102,103,104].

Hybrid dialysis strategies, such as combining PD with once-weekly hemodialysis, have also emerged as viable options. In Japan, this approach was associated with a 44% reduction in all-cause mortality compared to PD alone [105]. Additionally, incremental PD, a personalized regimen that begins with fewer exchanges and increases as residual renal function declines—has shown benefits in preserving kidney function, reducing the metabolic burden and infection risk, and lowering overall healthcare costs [106].

### 5.2. PD Solutions

Peritoneal dialysis (PD) solutions contain a mixture of electrolytes, buffering agents, and osmotic substances to enable effective solute and fluid exchange. Glucose remains the most widely used osmotic agent, and is available in various concentrations (1.36–4.25%) to generate ultrafiltration through osmotic gradients. However, the heat sterilization of glucose-based solutions leads to acidity and the accumulation of glucose degradation products (GDPs), reducing biocompatibility and potentially damaging the peritoneal membrane over time. To address these limitations, icodextrin—a 7.5% glucose polymer derived from starch, was developed as an alternative. Composed mainly of α-1,4 glycosidic bonds with minor α-1,6 linkages, icodextrin enables sustained ultrafiltration during long dwell times and belongs to the dextrin class of polysaccharides [107].

Recent efforts have focused on enhancing solution biocompatibility by formulating neutral pH, low-GDP solutions. Clinical evidence suggests these solutions better preserve the peritoneal membrane structure and reduce inflammatory responses [108]. Patients treated with neutral pH, low-GDP solutions show lower levels of proinflammatory cytokines such as TNF-α, IL-1, and IL-8 compared to those using conventional glucose-based fluids, supporting their improved biocompatibility [109]. A large 2023 study demonstrated significantly lower all-cause and cardiovascular mortality among patients using such solutions, although a slight increase in peritonitis rates was reported, warranting further research [103,105]. GDPs promote peritoneal fibrosis and neoangiogenesis via the activation of the RAGE pathway, which increases profibrotic (TGF-β, CTGF) and proangiogenic (VEGF) signaling. These mediators drive extracellular matrix accumulation, mesothelial-to-mesenchymal transition, and capillary proliferation [110].

Beyond improving pH and GDP profiles, novel solutions include exploring alternative osmotic agents such as icodextrin, xylitol, and L-carnitine. XyloCore ^®^, a new formulation partially substituting glucose with xylitol and L-carnitine, has shown promising results, including reduced fibrosis and inflammation in preclinical studies. The ongoing ELIXIR trial is evaluating its clinical safety and efficacy [111,112]. These agents offer advantages such as sustained ultrafiltration, improved peritoneal transport stability, and lower systemic glucose absorption compared to conventional glucose-based fluids. Preliminary evidence also suggests that xylitol-based solutions may positively influence systemic glucose metabolism and oxidative stress, although further studies are needed to clarify these effects [111,112].

Chronic exposure to conventional PD solutions with low pH and high GDPs induces persistent local immune activation involving neutrophils, macrophages, and T lymphocytes, promoting peritoneal fibrosis and membrane remodeling. This compromises dialysis membrane function and increases infection susceptibility. Biocompatible solutions, particularly those based on icodextrin or low-GDP formulations, may better preserve immune function and reduce infection-related complications. Improved biocompatible solutions reduce these immunological consequences, though long-term effects remain under study [107,113].

## 6. SARS-CoV-2 Infection in Patients on Peritoneal Dialysis

Early in the COVID-19 pandemic, concerns arose about potential viral transmission via PD effluent. However, a small prospective study found no detectable SARS-CoV-2 RNA in effluent samples from PD patients, suggesting that peritoneal fluid is unlikely to serve as a transmission route [114]. As the understanding of the disease evolved, attention soon shifted to clinical outcomes and risk factors among PD patients. DM, poorly controlled HTN, obesity and CVD were identified as key predictors of COVID-19-related mortality, reflecting trends observed in the general population [115].

A prospective study in Tunisia reported 8.4 infections per 1000 patient-months among 127 unvaccinated PD patients. Diabetes and glomerulonephritis were more frequent in infected individuals, though no significant differences were found in peritonitis, technique failure, or mortality between infected and non-infected groups. This supports PD as a safe modality during the pandemic, partly due to its home-based nature and telemedicine compatibility [116].

A Turkish multicenter study compared outcomes among PD, HD and non-dialysis patients with COVID-19 and found that PD patients had the highest rates of ICU admission and mortality. Although differences between PD and HD groups were not statistically significant, the trend suggested worse short-term outcomes in the PD group. Older age and elevated CRP levels were identified as key predictors of adverse outcomes [117]. A Turkish multicenter study also showed that PD patients had the highest combined ICU admission and mortality rate (33.3%) among matched groups, with elevated CRP and older age as key predictors [118].

A systematic review of 55 studies concluded that PD offered several advantages during the pandemic, such as minimized healthcare exposure and continuity of care via telehealth [119]. Unlike in-center hemodialysis, which involves regular hospital attendance and close contact with staff and other patients, peritoneal dialysis enables treatment at home, thereby reducing the risk of SARS-CoV-2 exposure and nosocomial infection. This logistical and infection control advantage contributed to PD being viewed as a safer modality during the pandemic [119].

Another study linked diabetes to poorer survival, while active vitamin D use (calcitriol) was associated with better outcomes, likely due to its immunomodulatory effects. Altered fibrinogen glycosylation and enhanced platelet aggregation may further increase thrombotic risk in this population [120]. Conversely, a higher number of vaccine doses and the use of corticosteroid therapy were associated with reduced mortality. Functional independence declined from 94% pre-infection to 63% during illness, highlighting the impact of COVID-19 on autonomy and the need for additional support during care [121].

A registry-based study reported a 28-day mortality of 13%, similar to HD, but worsened with Delta variant infections and poor functional status. Vaccination markedly improved survival, reducing 28-day mortality by ~30% per dose [122].

Emerging evidence has also demonstrated the presence of anti-SARS-CoV-2 IgG antibodies in the serum and peritoneal effluent of vaccinated PD patients. Individuals who received heterologous booster regimens showed significantly higher antibody titers and no documented reinfections during six months of follow-up, while reinfections occurred only among unvaccinated patients, albeit with mild clinical courses [123]. Italian data on 146 PD patients showed an infection incidence of 0.16 per patient-year. Among them, 22.2% of unvaccinated individuals died, while vaccinated patients had milder illness and no fatalities [124]. Additional evidence confirmed adequate antibody responses post-vaccination in PD patients, along with a reduced need for oxygen and fewer complications [125].

In summary, the reviewed studies illustrate the complex clinical course of COVID-19 in PD patients, where comorbidities significantly influence outcomes. While initial fears focused on viral presence in peritoneal fluid, PD has proven to be a safe option due to its home-based application and telemedicine adaptability. Prognostic markers such as diabetes, functional status, and calcitriol use are relevant to outcomes, but vaccination remains the most effective protective factor, dramatically reducing severity, hospitalization and mortality. Furthermore, the detection of anti-SARS-CoV-2 IgG in peritoneal effluent post-vaccination raises several important research questions: whether local antibody levels correlate with systemic immunity or clinical protection and whether effluent IgG could serve as a non-invasive biomarker for vaccine responsiveness in PD patients.

Table 1 provides a comparative overview of key clinical studies on COVID-19 in PD patients, including the impact of vaccination and prognostic factors.

## 7. Discussion

This review highlights the complex relationship between SARS-CoV-2, immune dysfunction, and vaccine response in peritoneal dialysis (PD) patients. Chronic kidney disease and common comorbidities such as diabetes, hypertension, and cardiovascular disease impair both innate and adaptive immunity, increasing vulnerability to severe COVID-19 and reducing vaccine efficacy. Still, studies show that PD patients can mount meaningful immune responses, especially after mRNA-based heterologous boosters.

The home-based nature of peritoneal dialysis reduced viral exposure during the pandemic and enabled continued care via telemedicine. Beyond infection prevention, telemedicine facilitated the early identification of complications and improved symptom tracking, particularly in settings with limited in-person access. When integrated with remote immunological monitoring tools, it may further enhance vaccine response assessment and support individualized booster strategies. However, additional infrastructure and standardized protocols are needed to optimize its use for longitudinal immune surveillance in this population. Vaccination is still central to protection. Early boosters, serological monitoring and the adjunctive use of calcitriol may enhance immune responses, particularly with mRNA vaccines, which show superior immunogenicity compared to inactivated or vector-based platforms.

PD patients also exhibit localized immunity, with anti-SARS-CoV-2 IgG detected in both serum and peritoneal effluent. Heterologous boosters yielded higher antibody titers and no reinfections within six months, while only unvaccinated patients experienced mild reinfections. These findings support individualized vaccine planning, immune monitoring, and telemedicine-guided care. Emerging evidence suggests that baseline lymphocyte profiles, CRP/IL-6, and cytokine markers may help identify patients who would benefit from early revaccination.

**Figure 3:**
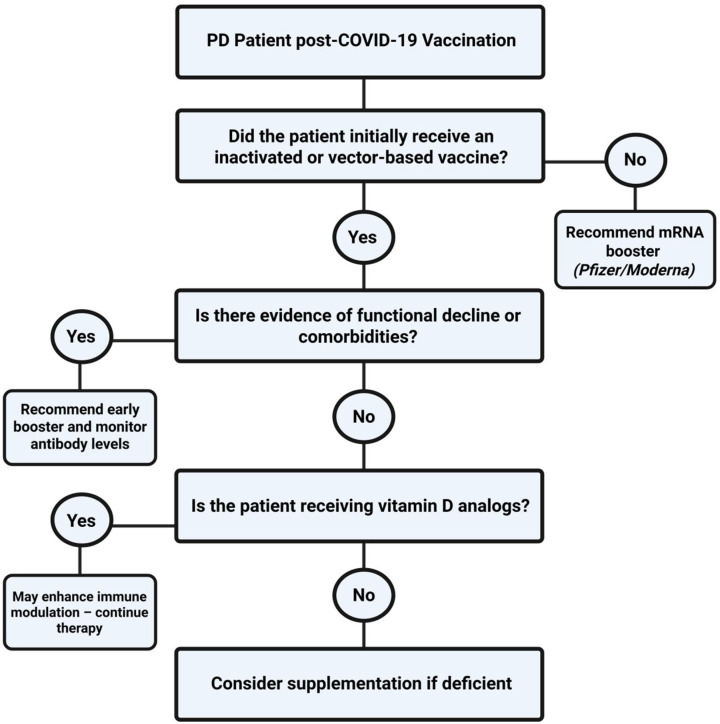
Suggested decision-making algorithm for optimizing SARS-CoV-2 booster strategies in PD patients based on initial vaccine type, functional status, and immunomodulatory therapy.

As outlined in Figure 3, structured decisions based on vaccine type, immune status, and ongoing therapies may optimize outcomes. In cases of poor response, passive immunization may be considered. While boosters remain essential, the long-term impact of repeated doses requires further study. Insights from PD patients may also guide care in other immunocompromised groups.

## 8. Public Health and Policy Considerations

The COVID-19 pandemic has shown the urgent need for inclusive public health strategies that adequately address the specific vulnerabilities of patients undergoing PD. Despite their high-risk status, PD patients have often been underrepresented in vaccine trials and national immunization plans [126]. Given the elevated mortality associated with comorbidities such as DM, HTN and CVD, frequent in this population, there is a compelling argument to prioritize PD patients in booster vaccination campaigns and variant-specific immunization strategies [127].

Equitable access to heterologous booster regimens, including mRNA-based vaccines, should be ensured, particularly in regions where initial vaccination involved inactivated or vector-based platforms with lower immunogenicity [128]. Policymakers must also recognize the protective value of home-based therapies like PD in reducing exposure to nosocomial transmission and should support the broader implementation of telemedicine infrastructure to sustain continuity of care [129]. Integrating dialysis-specific surveillance systems into national COVID-19 registries could enable the real-time tracking of vaccine responses, breakthrough infections and outcomes, ultimately informing more precise and individualized public health recommendations [130]. Ultimately, since PD and transplant patients may exhibit attenuated vaccine responses, they require ongoing monitoring to optimize immunization schedules and manage potential adverse effects, ensuring these immunocompromised populations receive tailored care [131].

## 9. Limitations of Current Evidence

Despite a growing body of literature on SARS-CoV-2 infection and vaccination in patients with chronic kidney disease, important limitations remain, especially regarding peritoneal dialysis. Many studies are observational in design, with small sample sizes and a limited statistical power, which reduces the generalizability of their findings. Data often come from single-center studies or national registries and may not reflect the global diversity in patient characteristics, vaccine types, or healthcare systems.

In addition, few studies compare outcomes between dialysis modalities, leaving uncertainty about whether immune responses and clinical results differ meaningfully between peritoneal and hemodialysis patients. The lack of standardized definitions for vaccine response, the inconsistent timing of antibody measurements, and methodological differences across studies make it difficult to interpret the immune dynamics. While some biomarkers like CRP, IL-6, and fibrinogen changes have been proposed as potentially useful, their predictive value and clinical relevance remain unconfirmed due to a lack of large, prospective studies.

These limitations highlight the need for well-structured, dialysis-specific research to guide future clinical decisions and improve outcomes in this vulnerable population.

## 10. Conclusions

The relationship between SARS-CoV-2 infection and peritoneal dialysis represents a clinically complex and immunologically vulnerable intersection that requires strategic, evidence-based approaches. Individuals undergoing peritoneal dialysis frequently demonstrate weakened immune responses, including reduced T-cell activity and short-lived, lower antibody titers, placing them at an elevated risk of severe disease progression. Despite these challenges, currently available vaccines have shown strong protective effects in this population. In particular, messenger RNA-based vaccines have demonstrated higher immunogenicity and better clinical outcomes compared to inactivated platforms. The administration of additional booster doses has proven beneficial in extending immune protection, especially when heterologous combinations are used.

From a practical standpoint, the vaccination of all peritoneal dialysis patients should be strongly prioritized, with heterologous booster schedules considered for individuals with functional decline, advanced age, or multiple comorbidities. When resources allow, assessing post-vaccination antibody titers may help identify patients with insufficient immune responses, who could benefit from intensified follow-up or alternative protective strategies. For patients with persistently weak humoral responses, passive immunization may offer adjunctive protection during high-risk periods. Adjunctive therapies with active vitamin D may also provide immune-supportive benefits and should be integrated into the broader management plan. The home-based nature of peritoneal dialysis, when supported by structured telemedicine protocols, offers an additional protective advantage by reducing nosocomial exposure and maintaining continuity of care during pandemics.

In conclusion, efforts to safeguard this population should focus on timely and individualized vaccination strategies, the strengthening of home-based care systems and the inclusion of targeted immunological support for high-risk individuals. Future research should aim to define optimal vaccination schedules, assess long-term immune durability and explore novel adjunctive therapies to reduce infection-related complications and improve outcomes in this uniquely susceptible group.

## Figures and Tables

**Table 1:** Comparative overview of key clinical studies evaluating SARS-CoV-2 infection in patients undergoing peritoneal dialysis.

Study/Author	Sample	Main Findings	Key Risk Factors	Vaccine Status	Outcome	Limitations
Candellier et al. (2020) [114]	10 PD patients	No SARS-CoV-2 RNA detected in PD effluent	Not applicable	Not reported	PD not a likely route of viral transmission	Very small sample; early pandemic stage
Badrouchi et al. (2022) [116]	127 unvaccinated PD patients	Incidence: 8.4 per 1000 pt-months. No significant differences in peritonitis, technique failure, or mortality	Male sex, diabetic nephropathy, glomerulonephritis	Unvaccinated	PD is safe and feasible during COVID-19	Single-center; small sample size; unvaccinated cohort limits generalizability
Abrahams et al. (2023) [117]	216 PD patients with COVID-19	3-month mortality: 40% (hospitalized), 37% (ICU); higher mortality than HD. 78% of survivors recovered fully	Hospitalization, ICU admission	Not reported	PD patients had worse outcomes than HD but good functional recovery in survivors	Observational; lack of detailed vaccine stratification
Kazancıoğlu et al. (2022) [118]	18 PD patients (compared to 18 HD and 18 controls)	Mortality 22.2% in PD; composite outcome (ICU/death) higher in PD (33.3%) than HD and controls	Older age, elevated CRP	Not reported	PD group had worse short-term outcomes than HD and control group	Small sample; retrospective design; limited generalizability
Painter et al. (2023) [119]	Systematic review	PD reduces healthcare contact; feasible with telemedicine	Not specified	Varied	PD effective and manageable with telemedicine	Heterogeneity of data; inclusion of case reports/series may introduce bias
Baralić et al. (2023) [120]	PD patients with COVID-19 (unspecified N)	Vitamin D linked to better survival. Calcitriol had immunomodulatory benefits	Diabetes, altered fibrinogen glycosylation	Not reported	Vitamin D may improve outcomes	No precise sample size; observational findings; no control group
Chuengsaman et al. (2022) [121]	Multicenter registry	28-day mortality = 13%. Vaccination lowered mortality by ~30% per dose	Functional impairment, Delta variant infection	Vaccinated/ unvaccinated compared	Vaccination improves survival	Registry-based; limited data on vaccination timing or comorbidity control
Baralić et al. (2024) [123]	28 PD patients	Anti-SARS-CoV-2 IgG detected in peritoneal effluent; higher levels in vaccinated	None reported	Sinopharm ± mRNA booster	No reinfection in vaccinated; mild reinfections in unvaccinated	Small sample; single center; short follow-up
Alfano et al. (2023) [124]	146 PD patients	COVID-19 incidence: 0.16/patient-year. Mortality: 22.2% (unvacc.), 0% (vacc.)	Vaccination status	Vaccinated vs. unvaccinated	Vaccination reduces severity and mortality	Single-center; retrospective; no randomized control group
Zheng et al. (2022) [125]	PD patients (unspecified N)	Vaccinated PD patients showed adequate antibody responses and fewer complications	Immunocompromised status	Vaccinated	Vaccines are effective in PD population	Sample size not specified; immune response only serologically assessed
